# Estimating causal effects with a *non-paranormal* method for the design of efficient intervention experiments

**DOI:** 10.1186/1471-2105-15-228

**Published:** 2014-06-30

**Authors:** Reiji Teramoto, Chiaki Saito, Shin-ichi Funahashi

**Affiliations:** 1 Department for Research, Forerunner Pharma Research, Co., Ltd, Yokohama, Japan

**Keywords:** Non-paranormal, Gaussian assumption, Causal effect, Intervention-calculus, Directed acyclic graph, Machine learning, Causal inference, Experiment design

## Abstract

**Background:**

Knockdown or overexpression of genes is widely used to identify genes that play important roles in many aspects of cellular functions and phenotypes. Because next-generation sequencing generates high-throughput data that allow us to detect genes, it is important to identify genes that drive functional and phenotypic changes of cells. However, conventional methods rely heavily on the assumption of normality and they often give incorrect results when the assumption is not true. To relax the Gaussian assumption in causal inference, we introduce the *non-paranormal* method to test conditional independence in the PC-algorithm. Then, we present the *non-paranormal* intervention-calculus when the directed acyclic graph (DAG) is absent (NPN-IDA), which incorporates the cumulative nature of effects through a cascaded pathway via causal inference for ranking causal genes against a phenotype with the *non-paranormal* method for estimating DAGs.

**Results:**

We demonstrate that causal inference with the *non-paranormal* method significantly improves the performance in estimating DAGs on synthetic data in comparison with the original PC-algorithm. Moreover, we show that NPN-IDA outperforms the conventional methods in exploring regulators of the flowering time in *Arabidopsis thaliana* and regulators that control the browning of white adipocytes in mice. Our results show that performance improvement in estimating DAGs contributes to an accurate estimation of causal effects.

**Conclusions:**

Although the simplest alternative procedure was used, our proposed method enables us to design efficient intervention experiments and can be applied to a wide range of research purposes, including drug discovery, because of its generality.

## Background

Intervention experiments, e.g., knockdown or overexpression, are commonly conducted to identify genes that determine cell fates such as differentiation [1], induction of pluripotency [2], and direct reprogramming [3]. Those experiments are now indispensable in biological and medical research. Although intervention experiments identify a causal gene responsible for a phenotype of interest, they are time-consuming and cost-intense. Therefore, it is very important to prioritize and focus on causal genes with high confidence. However, it is difficult to infer causal effects only from observational data. This task coincides with inferring causal effects that are established in Statistics. Note that in this problem setting, a causal effect is different from a regression-type effect of association [4]. In fact, previous studies suggested that representative regression methods such as lasso and elastic net are not appropriate for our goal [4-6].

Recently, there has been much progress to address this problem by employing the intervention-calculus when the directed acyclic graph (DAG) is absent (IDA) [4-6] for the design of efficient intervention experiments. IDA combines two methods: (1) inference the unknown underlying DAG model from observational data by the PC-algorithm [7] and (2) estimating causal effects on the basis of DAG using intervention-calculus; furthermore, it provides estimated lower bounds of total causal effects from observational data. The PC-algorithm is computationally feasible and appropriate for high-dimensional settings. In addition, it has the desirable property to achieve high computational efficiency as a function of sparseness of the true underlying DAG model.

In spite of these advantages, the PC-algorithm requires the Gaussianity assumption to use partial correlations to test conditional independence, and this assumption is not necessarily true in real data sets. Because the normality assumption is restrictive and conclusions inferred under this assumption could be misleading, it is desirable to relax the Gaussian assumption.

On the other hand, *non-paranormal* methods that use a semiparametric Gaussian copula have been proposed for estimating sparse undirected graphs and exhibit significant improvement in the performance because the normality assumption is relaxed [8,9]. The main idea of the *non-paranormal* method is to exploit the nonparametric correlation coefficient instead of Pearson’s correlation coefficient for estimation. Although this is the simplest alternative procedure, the *non-paranormal* graphical model could be a viable alternative for the Gaussian graphical model.

Consequently, we present *non-paranormal* IDA (NPN-IDA), which uses nonparametric partial correlations to test conditional independencies in the PC-algorithm for intervention-calculus. In our method, the Gaussian assumption in the PC-algorithm is naturally relaxed by using nonparametric partial correlation. Although the *non-paranormal* method has been successfully applied to estimating undirected graphs in previous studies, we show that it works well for estimating DAGs in the PC-algorithm. Next, we applied our method to *Arabidopsis thaliana* microarray data and mouse microarray data to demonstrate that NPN-IDA outperforms IDA in exploring regulators of the flowering time in *A. thaliana* and regulators that control the browning of white adipocytes in mice.

In summary, the three main contributions of this work are: (1) introduction of a *non-paranormal* method for inference of the unknown underlying DAG model from observational data in the expansive framework of the PC-algorithm, (2) combination of the *non-paranormal* method and the PC-algorithm significantly improves the performance in estimating DAGs on synthetic data, and (3) NPN-IDA is effective in exploring regulators that control specific phenotypes of interest.

## Methods

We first introduce the IDA procedure. IDA consists of (1) inference of the unknown underlying DAG model from observational data by PC-algorithm and (2) estimation of causal effects based on the DAG using intervention-calculus. Then, we introduce the *non-paranormal* method for PC-algorithm. Finally, we present the combination of the *non-paranormal* method for PC-algorithm and estimating causal effects as NPN-IDA algorithm.

### Inference DAGs with the PC-algorithm

**Figure 1:**
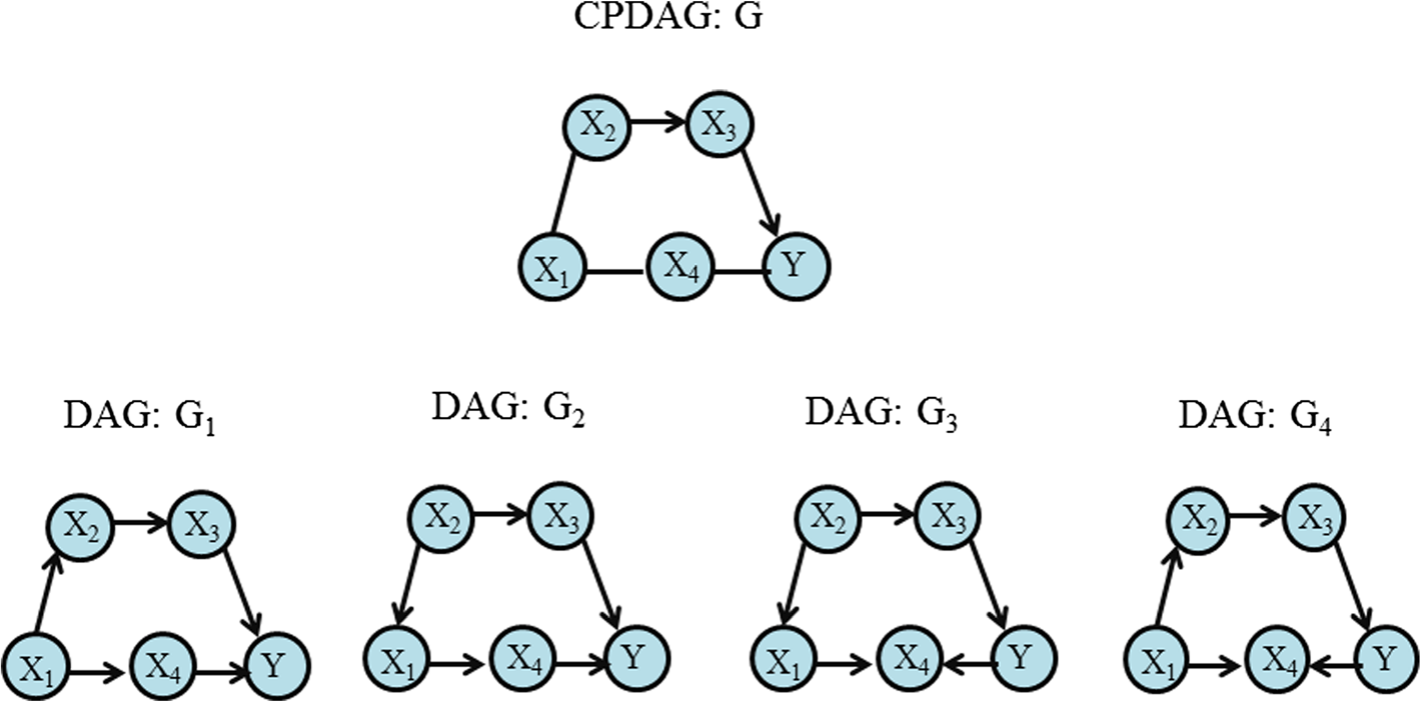
**Example of CPDAG.** CPDAG G with the DAGs G_1_, G_4_ that are in its equivalence class.

Let *G* = (*V*, *E*) be a graph consisting of vertices V and a set of edges *E* ⊆ *V* × *V*. In our context, the vertices represent random variables *X*_1_, …, *X*_*p*_, and Y. The edges represent relationships between pairs of these variables. It is possible that some DAGs fulfill the Markov condition and encode the same list of conditional independencies. Two DAGs are observationally equivalent only if they have the same skeleton and same sets of v-structures, i.e., two converging arrows whose tails are connected by an arrow. In this way, DAGs can be partitioned into equivalent classes, where all members are observationally equivalent and represent the same conditional independence. In a given conditional independence set of DAGs, one can only determine a DAG up to its equivalence class. The equivalence class is called completed partially directed acyclic graph (CPDAG). It has the same skeleton as every DAG in the equivalence class and directed edges only where all DAGs in the equivalence class have the same directed edge. Arrows that point into one direction for some DAGs in the equivalence class and in the other direction for other DAGs in the equivalence class are represented by undirected edges (Figure 1). By assuming that random variables are multivariate normally distributed, conditional independencies can be inferred from a partial correlation between *X*_*i*_ and *X*_*j*_ given a set of other variables S that equals zero:

(1) $$\begin{array}{ccc} \rho_{\mathrm{ij}|S}=\mathrm{Parcor}\left(X_{i}⊥X_{j}|X_{S}\right)=0 & \mathrm{iff} & X_{i}⊥X_{j}|X_{S} \end{array}$$

We then used the sample version of the PC-algorithm to estimate the corresponding CPDAG. This involves multiple testing for Fisher’s Z-transformed partial correlations,

$${\hat{Z}}_{\mathrm{ij}|S}=\frac{1}{2}\log\left(\frac{1+{\hat{\rho }}_{\mathrm{ij}|S}}{1-{\hat{\rho }}_{\mathrm{ij}|S}}\right).$$

Because ${\hat{Z}}_{\mathrm{ij}|S}$ has a *N*(0, (*n* − |*S*| − 3)^− 1^) distribution if *ρ*_*ij*|*S*_ = 0, we conclude that *ρ*_*ij*|*S*_ ≠ 0 if

$$\left|{\hat{Z}}_{\mathrm{ij}|S}\right|\sqrt{n-|S|-3}>\Phi^{-1}\left(1-\frac{\alpha }{2}\right),$$

where *Φ* is the standard normal distribution function and *α* is a tuning parameter, which can be interpreted as the significance level of a single partial correlation test. Choosing an appropriate value for *α* is difficult but, for example, can be done with the Bayesian information criterion.

**Figure 2:**
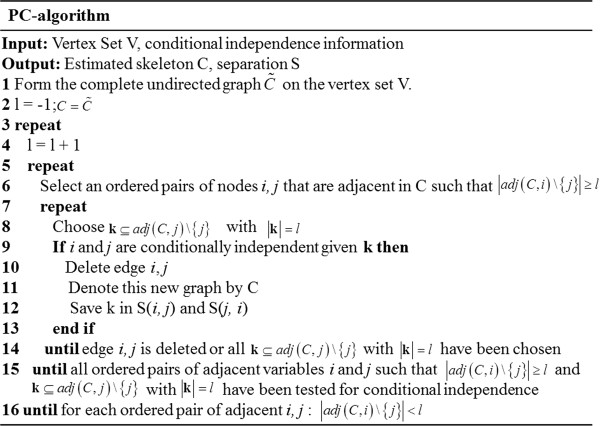
PC-algorithm for generating the skeleton.

First, the PC-algorithm generates a skeleton on the basis of conditional independencies. The outline of the PC-algorithm is shown in Figure 2. The complete PC-algorithm is described in detail in a precious work [7]. Note that the PC-algorithm employs partial correlation to test conditional independency.

### Estimating causal effects using intervention-calculus

Again, we considered p + 1 random variables *X*_1_, … *X*_*p*_, *Y* (also referred to as *X*_1_, … *X*_*p*_, *X*_*p* + 1_). Any distribution that is generated from a DAG with independent error is called Markovian with respect to the DAG. Therefore, any Markovian distribution can be factorized as

$$f\left(x_{1},\ldots ,x_{p+1}\right)=\prod_{j=1}^{p+1}f\left(x_{j}|pa_{j}\right).$$

To represent the effect of an intervention of a set of variables, a *do* operator is introduced. We denoted the distribution of Y that would occur if the treatment condition $X_{i}=x_{i}^{'}$ was enforced uniformly over the population via some intervention as $f\left(y|\mathrm{do}\left(X_{i}=x_{i}^{'}\right)\right)$. For a Markovian model, the distribution generated by an intervention $\mathrm{do}\left(X_{i}=x_{i}^{'}\right)$ on the set of variables *X*_1_, …, *X*_*p* + 1_ is given by the following truncated factorization formula:

(2) $$f\left(x_{1},\ldots ,x_{p+1}|\mathrm{do}\left(X_{i}=x_{i}^{'}\right)\right)=\left\{\begin{array}{c} \begin{array}{cc} \prod_{j=1,j\neq i}^{p+1}f\left(x_{j}|pa_{j}\right)|{}_{x_{i}=x_{i}^{'}}, & \begin{array}{cc} \mathrm{if} & x_{i}=x_{i}^{'} \end{array} \end{array}\\ \begin{array}{cc} 0, & \mathrm{otherwise}, \end{array} \end{array}\right)$$

Where *f*(*x*_*j*_|*pa*_*j*_) are the pre-intervention conditional distributions. Note that this formula employs the DAG structure to write the interventional distribution on the left-hand side in terms of pre-intervention conditional distributions on the right-hand side. The distribution of *Y* = *X*_*p* + 1_ after an intervention $\mathrm{do}\left(X_{i}=x_{i}^{'}\right)$ can be obtained by marginalizing out *x*_1_, …, *x*_*p*_ in equation (2). This can be written as follows:

$$f\left(y|\mathrm{do}\left(X_{i}=x_{i}^{'}\right)\right)=\left\{\begin{array}{c} \begin{array}{ccc} f\left(y\right), & \mathrm{if} & Y\in pa_{i}, \end{array}\\ \begin{array}{ccc} \int f\left(y|x_{i}^{'},pa_{i}\right)f\left(pa_{i}\right)\mathrm{dp}a_{i} & \mathrm{if} & Y\notin pa_{i}, \end{array} \end{array}\right)$$

where *f*(·) and $f\left(\overset{Ä}{n}|x_{i}^{'},pa_{i}\right)$ represent pre-intervention distributions. We can summarize the distribution generated by the intervention by its mean

$$E\left(Y|\mathrm{do}\left(X_{i}=x_{i}^{'}\right)\right)=\left\{\begin{array}{c} \begin{array}{ccc} E\left(Y\right), & \mathrm{if} & Y\in pa_{i} \end{array}\\ \begin{array}{ccc} \int E\left(Y|x_{i}^{'},pa_{i}\right)f\left(pa_{i}\right)\mathrm{dp}a_{i}, & \mathrm{if} & Y\notin pa_{i} \end{array} \end{array}\right)$$

and define the causal effect of $\mathrm{do}\left(X_{i}=x_{i}^{'}\right)$ on Y by

(3) $$\frac{\partial }{\partial x}E\left(Y|\mathrm{do}\left(X_{i}=x\right)\right)|{}_{x=x_{i}^{'}}$$

Under the assumption that *X*_1_, … *X*_*p*_, *Y* are jointly Gaussian, it is easy to compute the causal effects. Gaussianity implies that $E\left(Y|x_{i}^{'},pa_{i}\right)$ is linear in $x_{i}^{'}$ and *pa*_*i*_ That is

$$E\left(Y|x_{i}^{'},pa_{i}\right)=\gamma_{0}+\gamma_{i}x_{i}^{'}+\gamma_{pa_{i}}^{T}pa_{i}$$

for some values, *γ*_0_, *γ*_*i*_, and $\gamma_{pa_{i}}\in {\mathbb{R}}^{\left|pa_{i}\right|}$, where |*pa*_*i*_| is the cardinality of the set *pa*_*i*_. Then, we derive

$$\int E\left(Y|x_{i}^{'},pa_{i}\right)f\left(pa_{i}\right)\mathrm{dp}a_{i}=\gamma_{i}x_{i}^{'}+\int \gamma_{pa_{i}}^{T}pa_{i}f\left(pa_{i}\right)\mathrm{dp}a_{i},$$

which is linear in $x_{i}^{'}$. The causal effect of *X*_*i*_ on *Y* when *Y* ∉ *pa*_*i*_ can be computed with (3) yielding *γ*_*i*_, which is simply the regression coefficient of *X*_*i*_ in the regression of *Y* on *X*_*i*_ and *pa*_*i*_. When *Y* ∈ *pa*_*i*_, the causal effect becomes zero, because *Y* is a direct cause of *X*_*i*_. Therefore, the causal effect of *X*_*i*_ on *Y* is given by the following equation:

$$\beta_{i|pa_{i}}=\left\{\begin{array}{c} \begin{array}{ccc} 0, & \mathrm{if} & Y\in pa_{i} \end{array}\\ \begin{array}{ccc} \mathrm{coefficient}\;\mathrm{of}\;X_{i}\;\mathrm{in}\;Y∼X_{i}+pa_{i}, & \mathrm{if} & Y\notin pa_{i} \end{array} \end{array}\right),$$

where *Y* ∼ *X*_*i*_ + *pa*_*i*_ is a shorthand notation for the linear regression of *Y* on *X*_*i*_ and *pa*_*i*_. Consequently, in the Gaussian case, the causal effect does not depend on the value of $x_{i}^{'}$ and can be interpreted as

$$\beta_{i|pa_{i}}=E\left[Y|\mathrm{do}\left(X_{i}=x_{i}^{'}+1\right)\right]-E\left[Y|\mathrm{do}\left(X_{i}=x_{i}^{'}\right)\right]$$

for any value of $x_{i}^{'}$.

**Figure 3:**
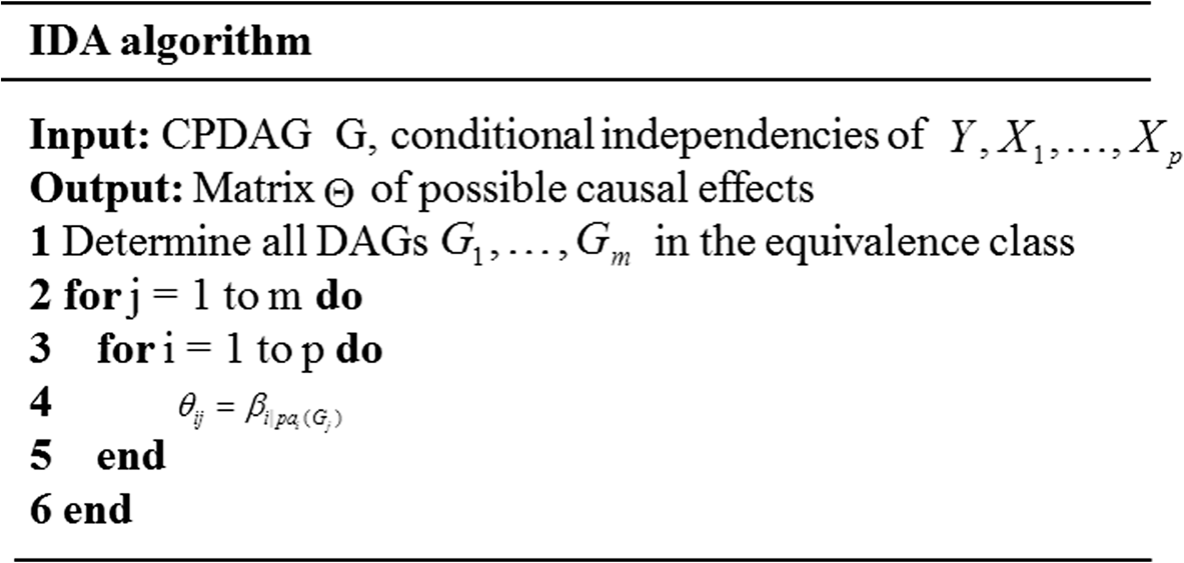
IDA algorithm.

Next, we will describe the IDA algorithm in detail. For simplicity, we only show the case in which the PC-algorithm gives the correct CPDAG. Details of the sample version of the IDA algorithm can be found in previously conducted studies [5-7]. On the basis of CPDAG, which is inferred from the PC-algorithm, we can compute the causal effect for every DAG in the equivalence class, which consists of multisets. Multisets differ from normal sets in that the multiplicity of the elements is taken into account. The IDA algorithm is given in Figure 3.

**Figure 4:**
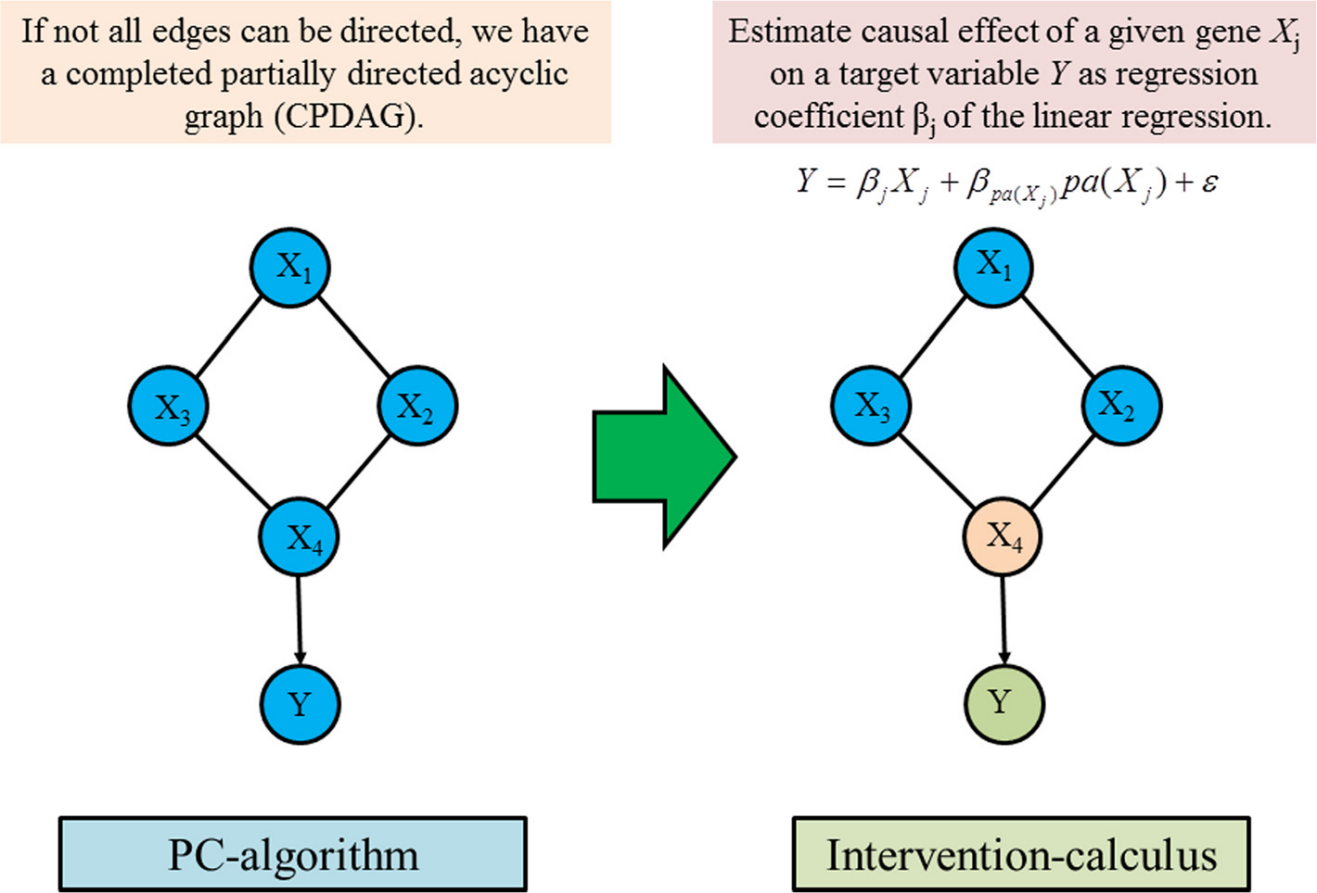
Illustrative procedure of IDA.

For computing the causal effects *θ*_*ij*_ of *X*_*i*_ on *Y* in DAG *G*_*j*_, the IDA algorithm simply computes the regression coefficient of *X*_*i*_ in a regression of *Y* on *X*_*i*_ and *pa*_*i*_, which is denoted by $\beta_{i|pa_{i}\left(G_{j}\right)}$. This procedure is performed for every DAG in the equivalence class yielding a vector of length *j* of possible causal effects, where *j* is the number of DAGs in the equivalence class. Computing the effect of every *X*_1_, …, *X*_*p*_ on *Y* yields a matrix *Θ* with *θ*_*ij*_ entries. We describe the illustrative procedure of IDA in Figure 4. When we obtain a single value for the estimated causal effect, we can take the minimal absolute value of the multiset as a lower bound from *Θ* for the true absolute intervention effect. This procedure intends to reduce the number of false positives. From a practical point of view, because the number of false positive should be kept as low as possible, it is desirable to provide conservative results.

### *Non-paranormal* method for NPN-IDA

In the PC-algorithm, the conditional independency is inferred from a sample partial correlation ${\hat{\rho }}_{\mathrm{ij}|S}$ between *X*_*i*_ and *X*_*j*_ given a set of other variables S. As described in the section of the PC-algorithm, a sample partial correlation coefficient can be obtained by calculating a sample Pearson’s correlation coefficient. This fact enables us to relax the Gaussianity assumption of the PC-algorithm by using the *non-paranormal* method. On the basis of [9], we derive the *non-paranormal* version of the PC-algorithm (NPN-PC algorithm). Let *f* = (*f*_1_, …, *f*_*p*_) be a set of monotonic univariate functions and let ∑ ^0^ ∈ *ℝ*^*p* × *p*^ be a positive definite correlation matrix with *diag*(∑^0^) = 1. We suppose that the *p*-dimensional random variable *X* = (*X*_1_, …, *X*_*p*_)^*T*^ has a *non-paranormal* distribution *X* ∼ *NPN*_*p*_(*f*, ∑ ^0^) if *f*(*X*) ≡ (*f*_1_(*X*_1_), …, *f*_*p*_(*X*_*p*_))^*T*^ ∼ *N*(0, ∑ ^0^). For continuous distributions, the *non-paranormal* family is equivalent to the Gaussian copula family [8]. It has been shown that the *non-paranormal* family is much richer than the normal family. The conditional independence is encoded by *Ω*^0^ = (∑^0^)^− 1^. Therefore, we can write the following formula given a set of other variables S:

(4) $$\begin{array}{ccc} \Omega_{\mathrm{ij}}^{0}=\rho_{\mathrm{ij}|s}=0 & \mathrm{iff} & X_{i}⊥X_{j}|X_{S} \end{array}$$

For Gaussian copula distributions, the following important lemma connects Spearman’s correlation coefficient $r_{\mathrm{ij}}^{s}$ to the underlying Pearson’s correlation coefficient [8,9].

**Lemma 1.**[10] By assuming *X* ∼ *NPN*(*f*, ∑ ^0^), we have $\sum_{\mathrm{ij}}^{0}=2\sin\left(\frac{\pi }{6}r_{\mathrm{ij}}^{s}\right)$.

According to the lemma, we can define the following estimator ${\hat{S}}^{\rho }=\left[{\hat{S}}_{\mathrm{ij}}^{\rho }\right]$ for the unknown correlation matrix ∑ ^0^: ${\hat{S}}_{\mathrm{ij}}^{\rho }=2\sin\left(\frac{\pi }{6}{\hat{r}}_{\mathrm{ij}}^{s}\right)$ for *i* ≠ *j*, and $\mathrm{diag}\left({\hat{S}}^{\rho }\right)=1$. Finally, if we define $p={\left({\hat{S}}^{\rho }\right)}^{-1}$, we obtain the following formula that connects Spearman’s correlation coefficient and the *non-paranormal* partial correlation coefficient $\rho_{\mathrm{ij}|s}^{s}$.

$$\rho_{\mathrm{ij}|S}^{s}=-\frac{p_{\mathrm{ij}}}{\sqrt{p_{\mathrm{ii}}p_{\mathrm{jj}}}}$$

**Figure 5:**
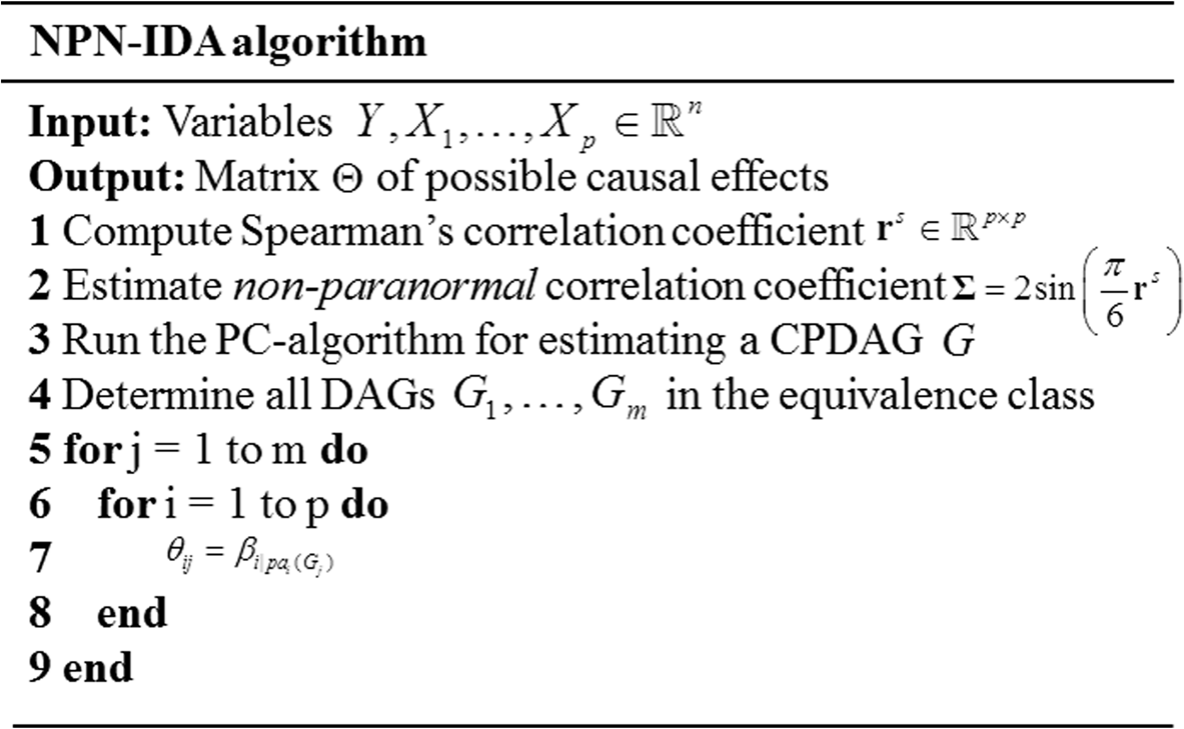
NPN-IDA algorithm.

Instead of *ρ*_*ij*|*S*_ in (1), we employ $\rho_{\mathrm{ij}|S}^{s}$ to test conditional independence for estimating CPDAG in the PC-algorithm. For simplicity, we denoted the method that combines the NPN-PC algorithm and IDA as NPN-IDA. We describe the pseudo code of the NPN-IDA algorithm in Figure 5.

## Results

### Experimental settings

Two experiments were conducted for different purposes. The first purpose was to evaluate the performance of the NPN-PC algorithm on synthetic data when the Gaussianity assumption is not true. According to a previous study [7], the four metrics of performance, i.e., true positive rate (TPR), false positive rate (FPR), true discovery rate (TDR), and structural hamming distance (SHD), are used. TPR, FPR, and TDR are defined as follows:

$$\mathrm{TPR}\;=\;\frac{\mathrm{Number}\;\mathrm{of}\;\mathrm{correctly}\;\mathrm{estimated}\;\mathrm{edges}}{\mathrm{Number}\;\mathrm{of}\;\mathrm{true}\;\mathrm{edges}},$$

$$\mathrm{FPR}\;=\;\frac{\mathrm{Number}\;\mathrm{of}\;\mathrm{incorrectly}\;\mathrm{estimated}\;\mathrm{edges}}{\mathrm{Number}\;\mathrm{of}\;\mathrm{true}\;\mathrm{gaps}},\;\mathrm{and}$$

$$\mathrm{TDR}\;=\;\frac{\mathrm{Number}\;\mathrm{of}\;\mathrm{correctly}\;\mathrm{estimated}\;\mathrm{edges}}{\mathrm{Number}\;\mathrm{of}\;\mathrm{estimated}\;\mathrm{edges}},$$

where gaps indicate the pairs of vertex that are not linked.

SHD is the number of edge insertions, deletions, and flips to transfer the estimated DAG into the true DAG [7]. A large SHD indicates a poor fit, whereas a small SHD indicates a good fit.

**Figure 6:**
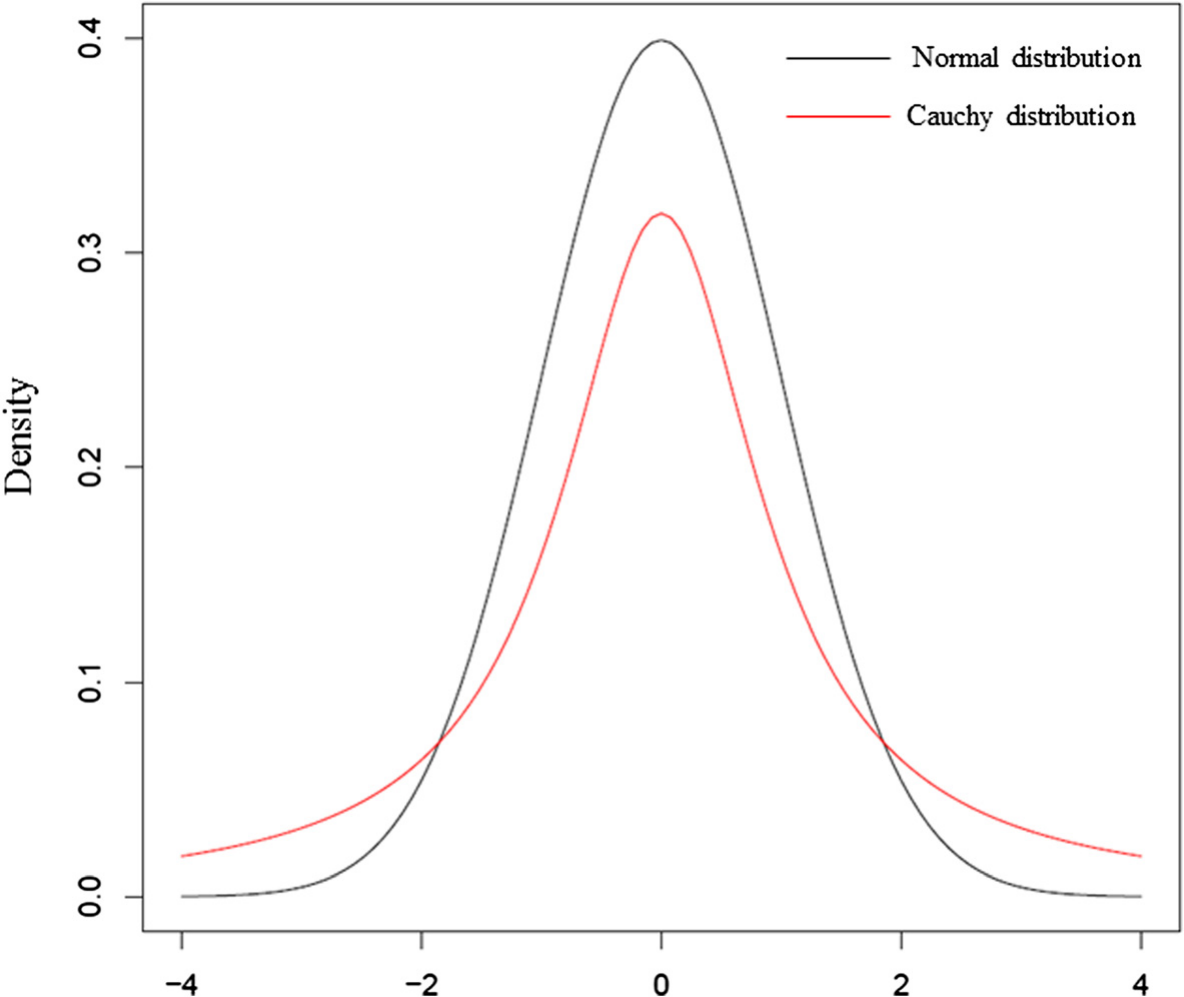
Comparison of standard normal distribution and standard Cauchy distribution.

To simulate the case that the Gaussian assumption is not true, we generated multivariate data with dependency structure by a given DAG with nodes corresponding to random variables and added standard Cauchy distribution in partial samples using the *rmvDAG* function in the *pcalg* package. Figure 6 depicts the normal distribution and the Cauchy distribution. As shown in Figure 6, the standard Cauchy distribution is tail-heavier than the standard normal distribution, and is quite different from the standard normal distribution.

The second purpose was to evaluate the performance of NPN-IDA when applied to predicting regulators of the flowering time as phenotype of interest using an *A. thaliana* microarray data set and regulators that control the browning of white adipocytes in mice using mouse microarray data. These two data sets are entirely different in terms of species and target variables. Therefore, if we obtain the consistent results thorough performance comparison of the methods, the consequence will be solid. We implemented the R language and employed the *pcalg* package for the PC-algorithm and IDA [11].

### Performance evaluation of the NPN-PC algorithm

**Figure 7:**
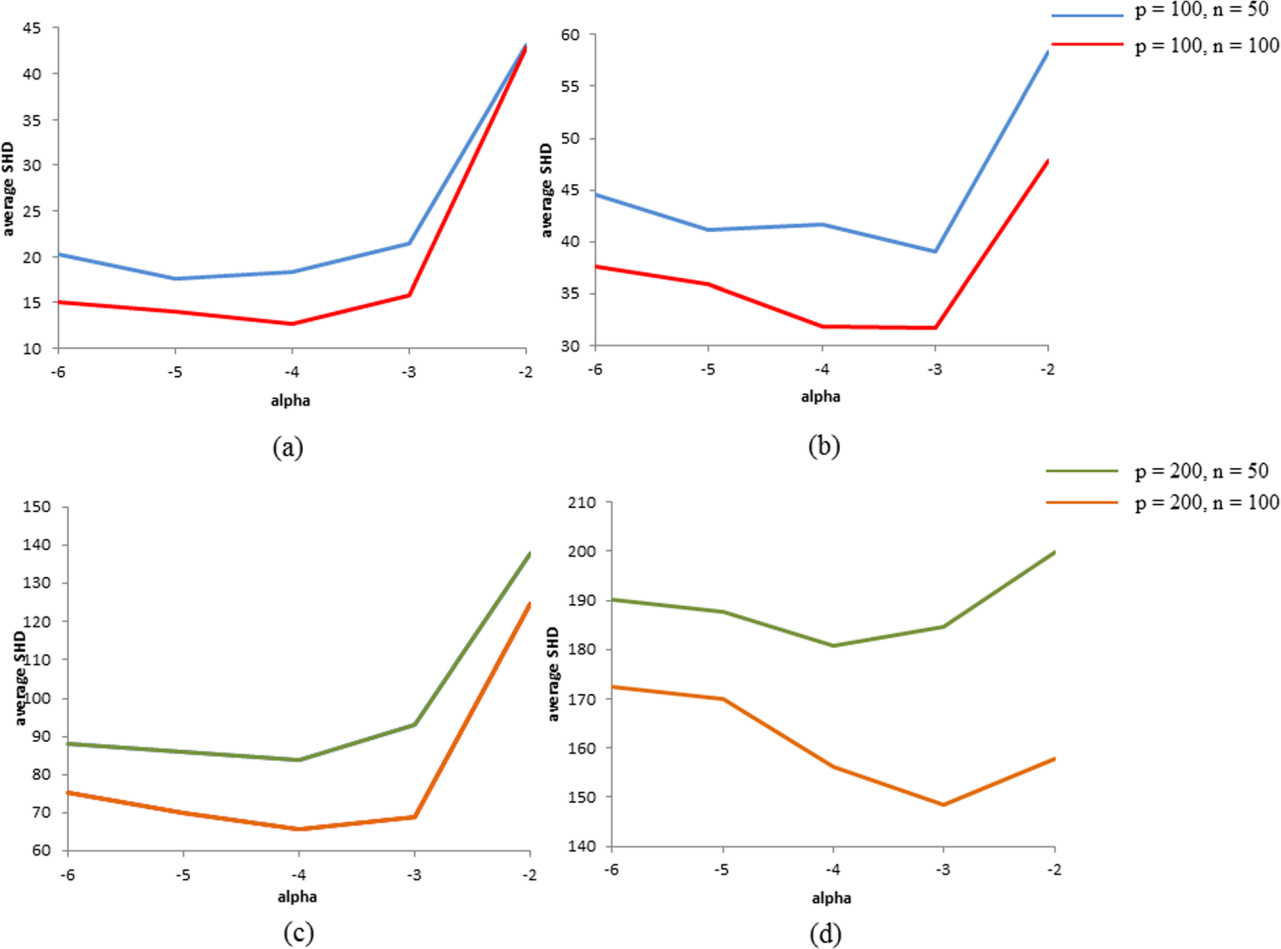
**Average SHD for p = 100 and 200 under different conditions of significance levels. (a) (c)** cp = 0.005, **(b) (d)** cp = 0.01.

We evaluated the performance under all combinations of settings listed in Table 1. The mixing rate indicates the percentage of samples whose error distribution was drawn from the standard Cauchy distribution. The higher the mixing rate, the less accurate is the Gaussianity assumption. To evaluate this hypothesis thoroughly, we repeated this experiment 50 times and averaged the values of TPR, FPR, TDR, and SHD. To explain the essence of these experiments, we show the representative results of the settings (*α* = 10^− 4^, cp = 0.005) in Table 2. All experimental results are shown in Additional file 1. To estimate the causal effects, estimated DAGs inferred by NPN-PC were employed. Because we considered the situation that the Gaussianity assumption is violated, we determined the significance level *α* on the basis of the average SHD when the mixing rate was 1. Average SHDs under the different probabilities of connecting one node to another node are shown in Figure 7. Because the average SHD was very small, we employed estimated DAGs when the significance level *α* was set to 10^−4^ to further estimate the causal effects.

**Table 1:** Parameter setting for performance evaluation of the NPN-PC algorithm

**Parameters**	**Set values**
α	10^−6^,10^−5^, 10^−4^, 10^−3^, 10^−2^
p	100, 200
n	50, 100
cp	0.005, 0.01
Mixing rate	0.1, 0.2, 0.3, 0.4, 0.5, 0.6, 0.7, 0.8, 0.9, 1

α: significant level, p: number of variables, n: number of samples, cp: probability of connecting one node to another node.

**Table 2:** Performance comparison between PC-algorithm and NPN-PC algorithm

**p**	**n**	**Mixing rate**	**TPR**		**FPR**		**TDR**		**SHD**	
			**PC**	**NPN-PC**	**PC**	**NPN-PC**	**PC**	**NPN-PC**	**PC**	**NPN-PC**
100	50	0.1	0.360	**0.373**	0.0021	**0.0003**	0.468	**0.876**	28.72	**19.40**
		0.2	0.341	**0.395**	0.0035	**0.0002**	0.326	**0.899**	35.74	**18.42**
		0.3	0.316	**0.372**	0.0044	**0.0002**	0.263	**0.898**	40.08	**18.10**
		0.4	0.325	**0.387**	0.0046	**0.0003**	0.266	**0.886**	42.58	**19.88**
		0.5	0.308	**0.393**	0.0047	**0.0002**	0.257	**0.908**	43.30	**18.92**
		0.6	0.326	**0.399**	0.0049	**0.0002**	0.252	**0.890**	42.88	**18.68**
		0.7	0.310	**0.409**	0.0051	**0.0002**	0.238	**0.893**	45.14	**18.80**
		0.8	0.297	**0.405**	0.0052	**0.0002**	0.221	**0.898**	44.64	**18.04**
		0.9	0.319	**0.432**	0.0052	**0.0002**	0.237	**0.903**	44.64	**17.74**
		1	0.313	**0.416**	0.0051	**0.0002**	0.241	**0.904**	44.74	**18.24**
	100	0.1	0.487	**0.625**	0.0031	**0.0001**	0.440	**0.956**	31.38	**13.94**
		0.2	0.451	**0.623**	0.0048	**0.0002**	0.331	**0.954**	42.30	**15.04**
		0.3	0.457	**0.653**	0.0058	**0.0001**	0.282	**0.963**	46.04	**13.60**
		0.4	0.442	**0.643**	0.0063	**0.0002**	0.265	**0.942**	48.84	**14.14**
		0.5	0.425	**0.667**	0.0064	**0.0001**	0.249	**0.965**	49.50	**12.90**
		0.6	0.449	**0.642**	0.0064	**0.0002**	0.264	**0.950**	49.32	**13.92**
		0.7	0.454	**0.681**	0.0064	**0.0002**	0.274	**0.951**	50.16	**13.50**
		0.8	0.421	**0.665**	0.0064	**0.0002**	0.257	**0.957**	49.78	**13.68**
		0.9	0.437	**0.692**	0.0064	**0.0001**	0.254	**0.965**	48.80	**12.30**
		1	0.401	**0.672**	0.0064	**0.0002**	0.239	**0.950**	50.00	**12.66**
200	50	0.1	0.291	**0.315**	0.0011	**0.0002**	0.564	**0.894**	105.72	**86.50**
		0.2	0.268	**0.326**	0.0018	**0.0002**	0.430	**0.892**	120.72	**85.08**
		0.3	0.256	**0.323**	0.0021	**0.0002**	0.386	**0.906**	127.88	**85.28**
		0.4	0.257	**0.317**	0.0023	**0.0002**	0.366	**0.903**	130.22	**84.86**
		0.5	0.255	**0.324**	0.0023	**0.0002**	0.360	**0.897**	132.10	**86.32**
		0.6	0.258	**0.345**	0.0024	**0.0002**	0.347	**0.885**	129.06	**81.48**
		0.7	0.249	**0.335**	0.0024	**0.0002**	0.345	**0.908**	131.80	**82.98**
		0.8	0.243	**0.335**	0.0024	**0.0002**	0.343	**0.888**	134.52	**85.38**
		0.9	0.250	**0.338**	0.0024	**0.0002**	0.344	**0.896**	133.46	**83.98**
		1	0.249	**0.340**	0.0024	**0.0002**	0.350	**0.903**	133.42	**83.94**
	100	0.1	0.424	**0.542**	0.0018	**0.0001**	0.534	**0.956**	110.60	**68.40**
		0.2	0.387	**0.565**	0.0027	**0.0001**	0.415	**0.968**	131.66	**66.50**
		0.3	0.374	**0.558**	0.0031	**0.0001**	0.377	**0.964**	140.38	**68.48**
		0.4	0.365	**0.567**	0.0032	**0.0001**	0.368	**0.968**	145.72	**68.72**
		0.5	0.372	**0.580**	0.0033	**0.0001**	0.357	**0.963**	142.34	**64.52**
		0.6	0.360	**0.576**	0.0034	**0.0001**	0.349	**0.967**	146.92	**66.90**
		0.7	0.363	**0.577**	0.0033	**0.0001**	0.354	**0.965**	146.84	**67.20**
		0.8	0.363	**0.581**	0.0034	**0.0001**	0.346	**0.968**	146.18	**65.90**
		0.9	0.372	**0.585**	0.0034	**0.0001**	0.361	**0.964**	145.62	**67.04**
		1	0.369	**0.594**	0.0034	**0.0001**	0.356	**0.972**	145.32	**65.72**

The bold indicates the best performance.

### Performance evaluation of the NPN-IDA algorithm

#### Exploring regulators of the flowering time in *A. thaliana*

**Figure 8:**
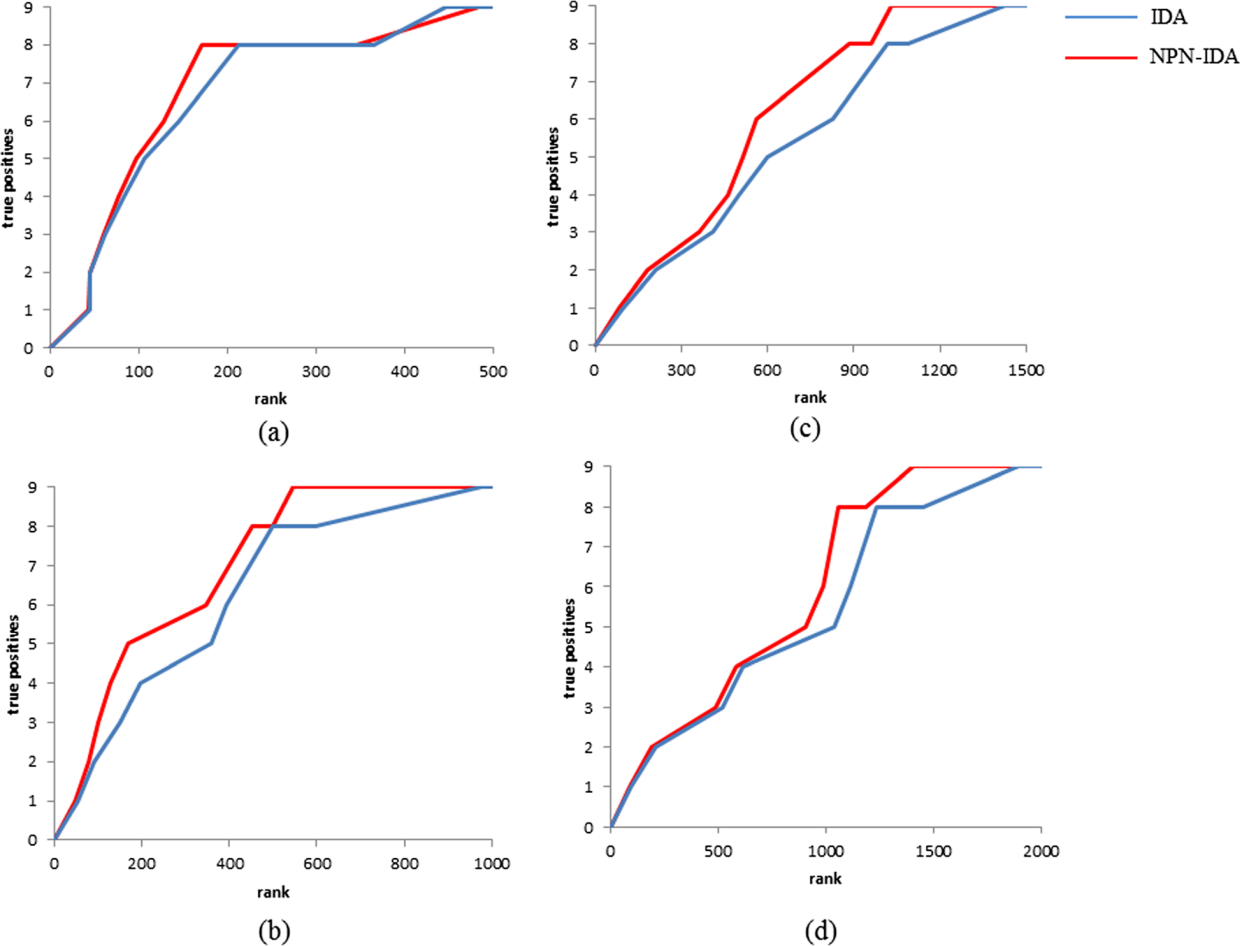
**Enrichment curves on exploring regulators of the flowering time in *Arabidopsis thaliana. *(a)** Correlation screening is set to 500, **(b)** correlation screening is set to 1000, **(c)** correlation screening is set to 1500, and **(d)** correlation screening is set to 2000.

We employed the microarray data set of *A. thaliana* and the flowering time used in a previous study [7]. The data set consisted of 21326 probes and 47 samples. We regarded the nine known regulators of the flowering time (*DWF4*, *FLC*, *FRI*, *RPA2B*, *SOC1*, *PDH-E1 BETA*, *ATGH9B5*, *LRR*, and *OTLD1*) as true-positive genes. Because IDA requires a significant amount of computation time, we selected genes for further estimation that had higher absolute correlation coefficients against flowering time until a predefined number was reached (correlation screening). In this study, the numbers of remaining genes obtained by correlation screening were set to 500, 1000, 1500, and 2000. According to the description in the previous section, we set the significance level *α* to 10^−4^ for estimating DAGs. Figure 8 shows the enrichment curves under the different conditions of correlation screening. Vertical axes represent the average numbers of true positives and horizontal axes indicate the ranks of causal effects. To quantitatively compare the performances of IDA and NPN-IDA, we also summarized the area under the enrichment curves (AUCs) under the different conditions of correlation screening in Table 3.

**Table 3:** **AUC values under the different conditions of correlation screening on exploring regulators of the flowering time in *Arabidopsis thaliana***

**Correlation screening**	**IDA**	**NPN-IDA**
500	0.737	**0.755**
1000	0.696	**0.776**
1500	0.605	**0.680**
2000	0.608	**0.662**

The bold indicates the best performance.

### Exploring the regulators that control the browning of white adipocytes in mice

We employed the mouse microarray data set of white adipose tissue (WAT) obtained from a previous study [12]. The data set consisted of 43681 probes and 349 WAT samples. According to a previous review [13], we regarded *Ucp1* as marker of brown adipose tissue (BAT). We selected genes that had higher absolute correlation coefficients against *Ucp1* until a predefined number was reached. The numbers of remaining genes were set to 2000, 3000, and 4000. Table 4 shows the known regulators of the differentiation of WAT to BAT for the different conditions of correlation screening. Note that there are no true positive genes when the number of remaining genes obtained by correlation screening is below 1000.

**Table 4:** Known regulators of the differentiation of WAT to BAT

**Correlation screening**	**Gene symbol**
2000	*Ppargc1a*, *Tbx15*, *Tfam*, *Bmp7*
3000	*Ppargc1a*, *Tbx15*, *Tfam*, *Bmp7*
4000	*Foxc2*, *Ppargc1a*, *Tbx15*, *Tfam*, *Bmp7*

**Figure 9:**
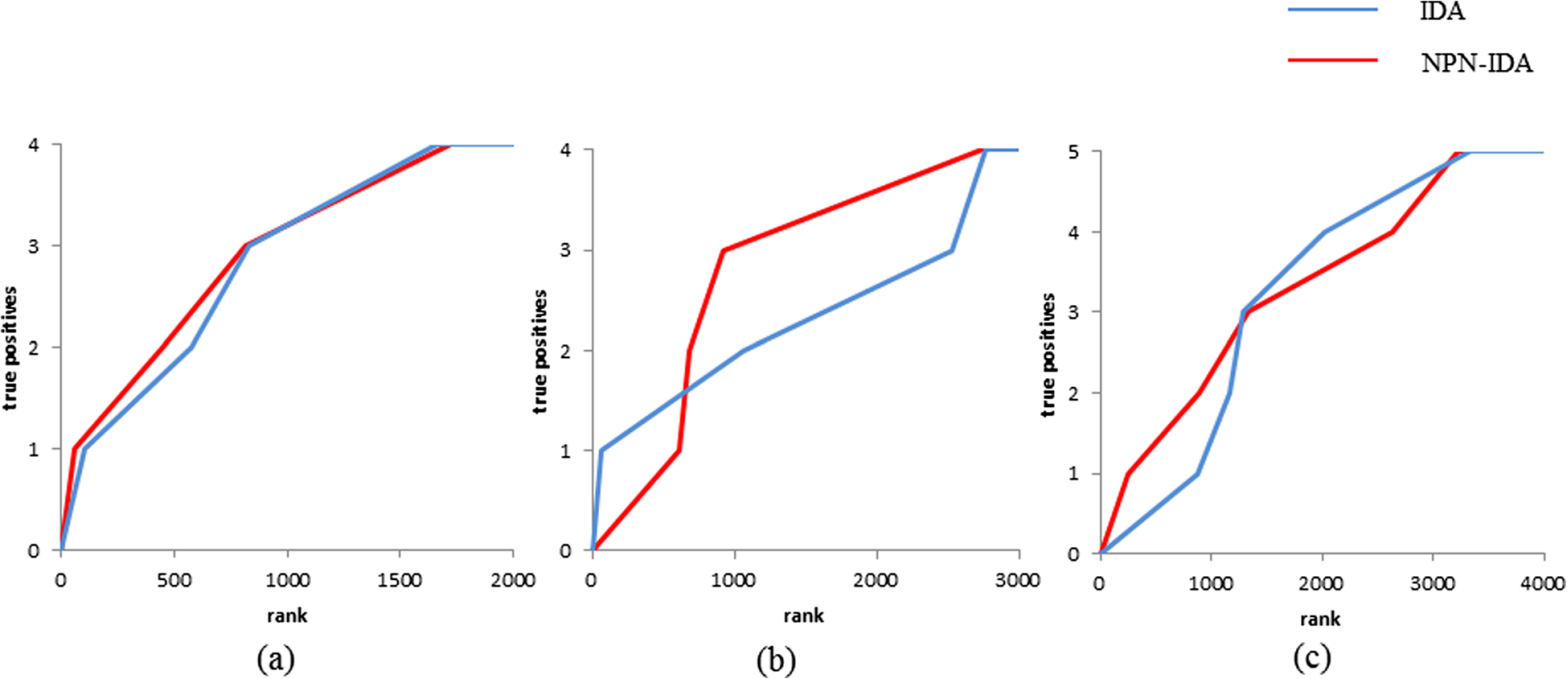
**Enrichment curves on exploring the regulators that control the browning of white adipocytes. (a)** Correlation screening is 2000, **(b)** correlation screening is 3000, and **(c)** correlation screening is 4000.

Because there are two probes for Tbx15, i.e., merck-NM_009323_at and merck-NM_01154_a_at, we regarded the probe that had the larger causal effect as Tbx15. Figure 9 shows the enrichment curves under different conditions of correlation screening. Vertical axes represent the average numbers of true positives and horizontal axes indicate the ranks of causal effects. The AUCs under the different conditions of correlation screening are summarized in Table 5.

**Table 5:** AUC values under the different conditions of correlation screening on exploring the regulators that control the browning of white adipocytes in mice

**Correlation screening**	**Known regulators**	**IDA**	**NPN-IDA**
2000	4	0.706	**0.726**
3000	4	0.580	**0.701**
4000	5	0.648	**0.665**

The bold indicates the best performance.

## Discussion

From Table 2, it appears that the NPN-PC algorithm consistently outperforms the PC-algorithm with regard to all performance metrics, TPR, FPR, TDR, and SHD. Furthermore, as the mixing rate increases, the performance values of the PC-algorithm decrease. This result clearly shows that the PC-algorithm does not work when the Gaussianity assumption is not true. In contrast, the NPN-PC algorithm works well when mixing rate is high. In other words, the NPN-PC algorithm does not require the Gaussianity assumption of data distribution. In terms of FPR, it appears that the FPR of the NPN-PC algorithm is strictly suppressed under all set values. In the NPN-PC algorithm, all performance metrics improved when the number of samples was 100 compared to when the number of samples was 50. From these observations, it can be concluded that the NPN-PC algorithm is robust and does not depend on data distribution, unlike the PC-algorithm. This characteristic is very attractive from the practical point of view.

From Table 3 and Figure 8, NPN-IDA consistently outperformed IDA on exploring regulators of the flowering time in *A. thaliana*. In particular, when the correlation screenings were set to 1000 and 1500, the difference in the AUCs between NPN-IDA and IDA increased. When the correlation screenings were set to 500, both NPN-IDA and IDA worked well. This result indicates that the known regulators were sufficiently recovered against the flowering time within genes that have high absolute correlation coefficients. Therefore, although we do not know whether the unknown regulators have high absolute correlation coefficients against the flowering time, it would be a good strategy from a practical perspective to explore novel regulators from genes that have high absolute correlation coefficients against the flowering time.

From Table 5 and Figure 9, NPN-IDA consistently outperformed IDA on exploring regulators that control the browning of white adipocytes in mice. In particular, when the correlation screenings were set to 3000 and 4000, the difference in the AUCs between NPN-IDA and IDA increased. These results suggest that NPN-IDA successfully enriches known regulators at the top ranks when the number of available genes increases. Consequently, our two experiments clearly demonstrated that NPN-IDA performs better than IDA.

**Figure 10:**
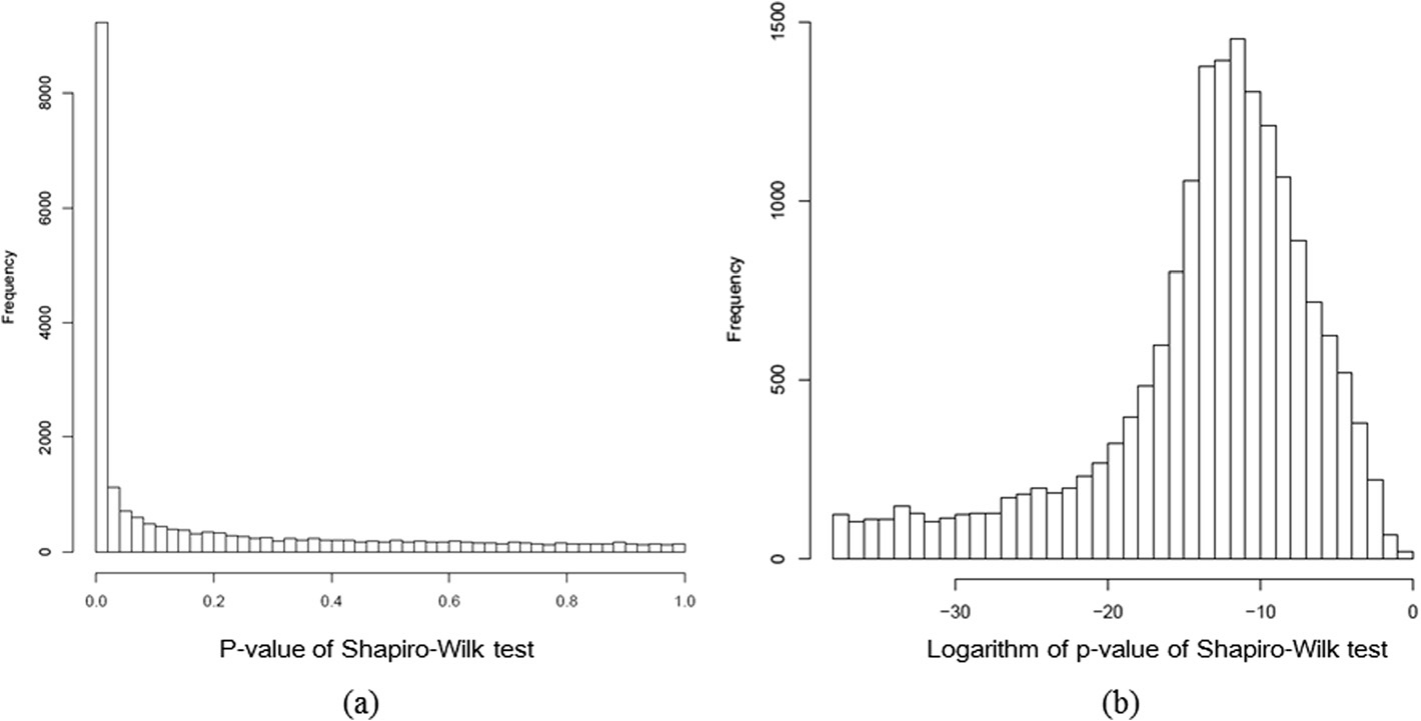
**Histogram of p-values of the Shapiro-Wilk test. (a)***Arabidopsis thaliana* microarray data and **(b)** mouse WAT microarray data (logarithmic scale).

**Figure 11:**
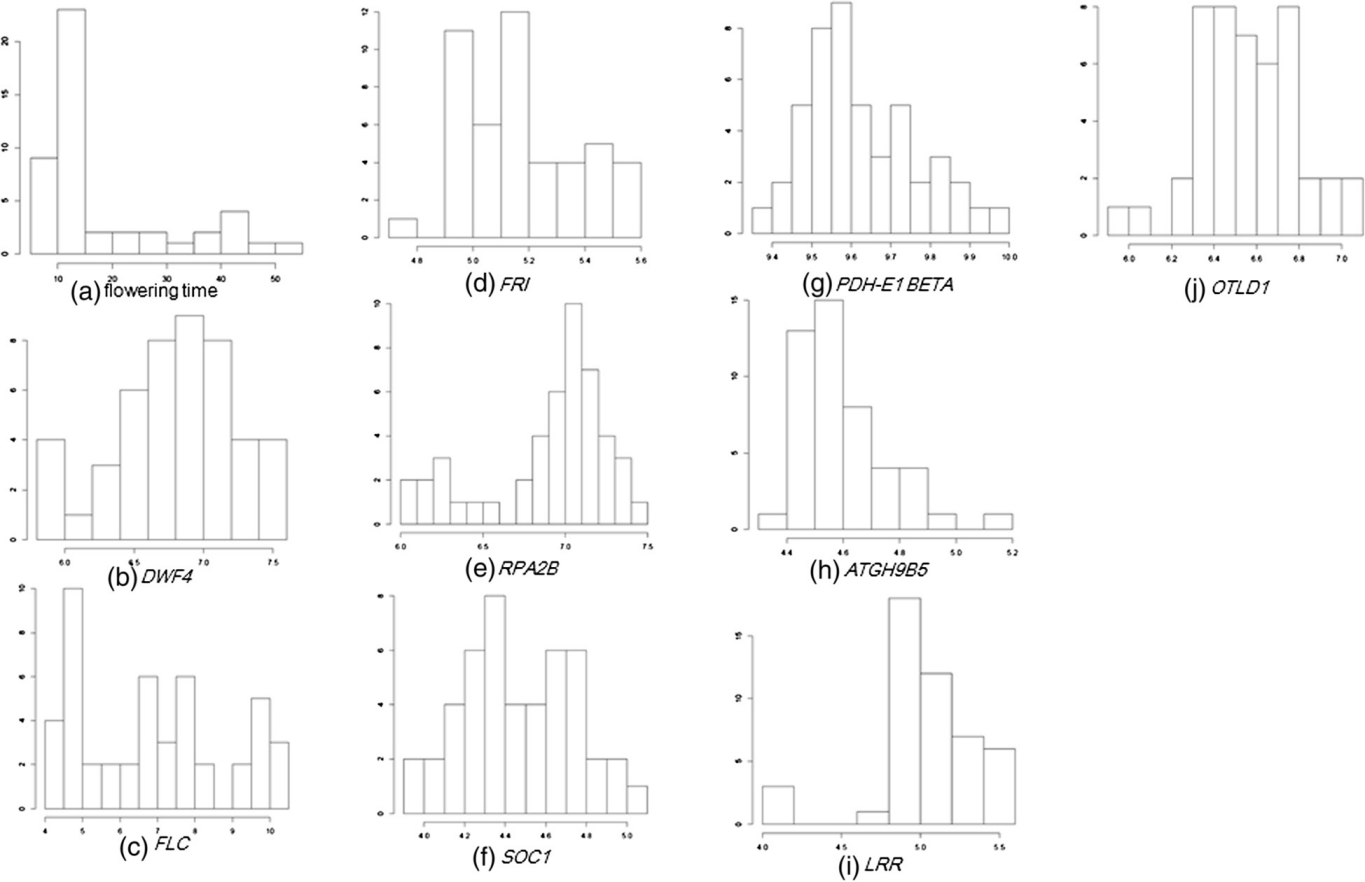
**Distributions of flowering time and expression levels of the nine known regulators of the flowering time.** Each panel is a histogram of the flowering time and each gene’s expression.

**Figure 12:**
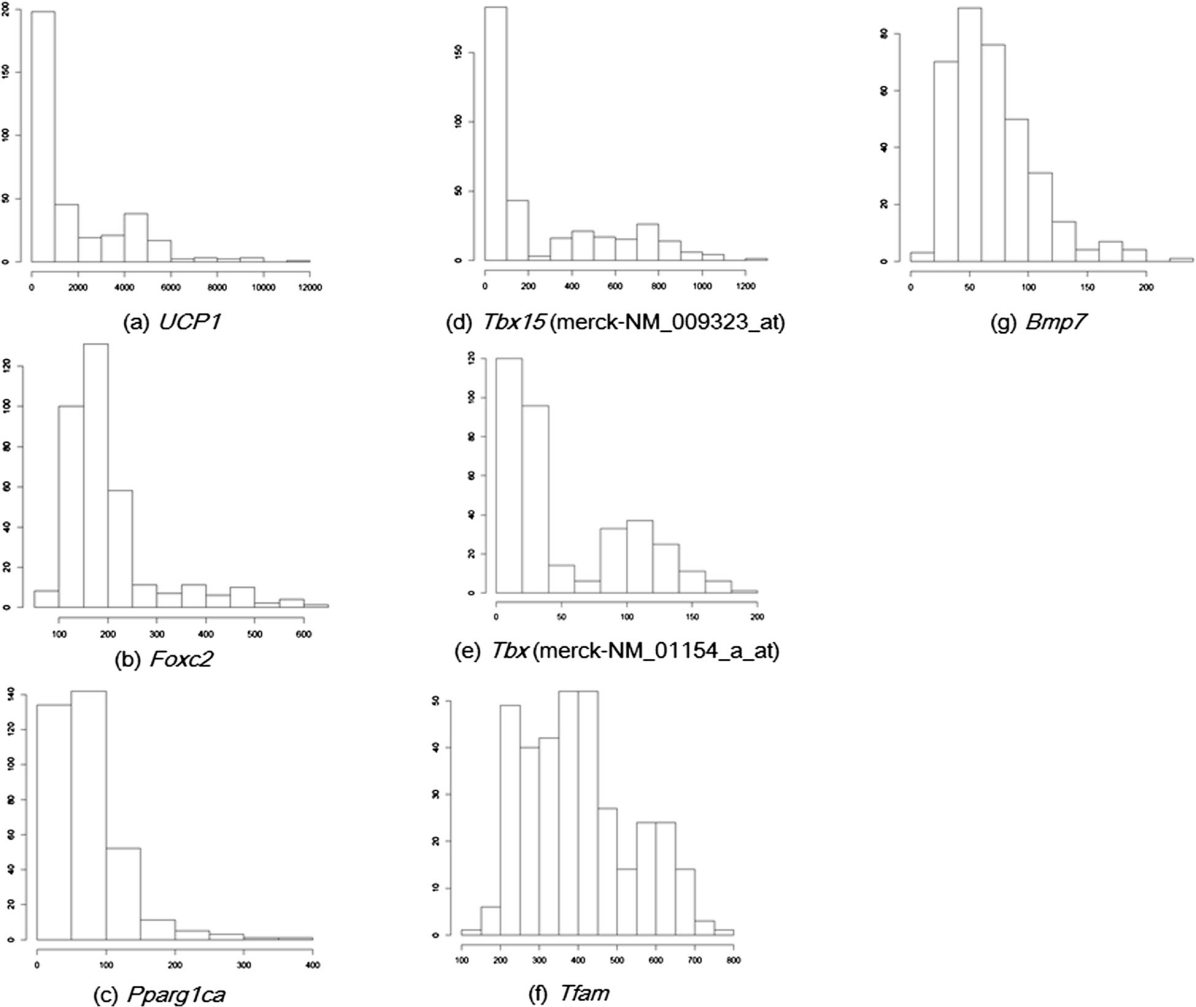
**Distributions of expression levels of the BAT marker gene *Ucp1 *and the five known genes.***Tbx* has two different probes. Each panel is a histogram of each gene’s expression.

To discuss whether the Gaussian assumption is true or not in the data sets used in this study, we applied the Shapiro-Wilk test to microarray data and a target variable of interest, which tests the null hypothesis that a sample came from a normally distributed population [14]. We show a histogram of the p-values of the Shapiro-Wilk test for target phenotypes of interest (flowering time in *A. thaliana* and gene expression of *Ucp1* in mice) and gene expression levels in Figure 10. We also show individual histograms of phenotypes of interest and expression levels of the known regulators in Figure 11; the respective p-values of the Shapiro-Wilk test are summarized in Table 6. From Figure 10, it appears that the p-values of most genes were <0.05 for both *A. thaliana* and mouse WAT microarray data. In other words, the normality assumption was violated in most genes. These results justify the use of NPN-IDA rather than IDA, because the latter method requires a normal distribution. With regard to the *A. thaliana* data, it appears that the p-values for flowering time, *FLC*, *FRI*, *RPA2B*, *ATGH9B5*, and *LRR* were <0.05 (Table 6); as shown in Figure 11, their distributions were skewed. With regard to the mouse WAT data, the p-values of all genes were very small (Table 6). As shown in Figure 12, their distributions were also skewed. These results strongly suggest that NPN-IDA, which does not require the Gaussian distribution, works better than IDA.

**Table 6:** p-values of the Shapiro-Wilk test of target variables and known regulators

**Variable**	**p-value**
Flowering time	1.09e-7
*DWF4*	3.62e-1
*FLC*	1.81e-3
*FRI*	2.81e-1
*RPA2B*	1.24e-4
*SOC1*	6.86e-1
*PDH-E1 BETA*	7.95e-2
*ATGH9B5*	1.69e-4
*LRR*	1.21e-4
*OTLD1*	6.84e-1
*Ucp1*	1.50e-21
*Foxc2*	6.01e-22
*Ppargc1a*	8.59e-21
*Tbx15* (*merck-NM_00923_at*)	1.75e-21
*Tbx15* (*merck-NM_01154_a_at*)	6.31e-20
*Tfam*	5.12e-08
*Bmp7*	3.09e-13

Although the method presented here performs significantly better than IDA, one might consider the difference in the performance as too small. However, within the known regulators of flowering time in *A. thaliana*, four genes (*PDH-E1 BETA*, *ATGH9B5*, *LRR*, and *OTLD1*) were experimentally validated using the IDA-based method [6]. Therefore, one should take into account that some of the known regulators were obtained on the basis of the Gaussian assumption. Thus, it is possible that the improvement achieved with NPN-IDA is greater than the experimental results shown in this study.

In summary, the two main results of our experimental study are: (1) the NPN-PC algorithm works better than the PC-algorithm in estimating DAGs on synthetic data, and (2) NPN-IDA performs better than does IDA in predicting regulators of the flowering time in *A. thaliana* and regulators of the differentiation of WAT to BAT in mice on the basis of microarray data. From these two results, we conclude that the performance to estimate DAGs contributes to the accurate prediction of causal effects.

From a practical point of view, because regulators that control the browning of white adipocytes have not been identified only from microarray data of WAT so far, it might be worthwhile to demonstrate this possibility for the first time using our novel method.

For further performance enhancement, we consider that NPN-IDA could be embedded into CStaR (causal stability ranking) [6] in the future. CStaR uses IDA with stability selection [6,15] and the performance was greatly improved compared to plain IDA. By simply replacing IDA with NPN-IDA in the estimation process for causal effects, it would be easy to combine NPN-IDA with CStaR.

## Conclusions

We presented NPN-IDA, which uses nonparametric partial correlations, to test conditional independencies in the PC-algorithm for intervention-calculus. To reveal the contribution of estimating DAGs, we evaluated the NPN-PC algorithm to estimate DAGs with the *non-paranormal* method. The two main results of our experimental study are: (1) the NPN-PC algorithm works better than the PC-algorithm in estimating DAGs on synthetic data, and (2) NPN-IDA performs better than IDA does in predicting regulators of the flowering time in *A. thaliana* and regulators of the differentiation of WAT to BAT in mice. Considering these two results, we concluded that the performance to estimate DAGs contributes to the accurate prediction of causal effects.

Thus, the simplest alternative procedure we used for our proposed method enables us to design efficient intervention experiments and can be applied to a wide range of research purposes, including drug discovery and medicine, because of its generality.

### Availability of supporting data

The microarray data sets used in this study are deposited in Additional file 2.

## Competing interests

The authors declare that they have no competing interests.

## Authors’ contributions

RT proposed the method, implemented, conduct experiments and wrote the manuscript. CS edited the tables. SF supervised the research project. All authors read and approved the final manuscript.

## Supplementary Material

Additional file 1 Experimental results of all combined parameter settings for performance comparison between the NPN-PC algorithm and the PC-algorithm. Click here for file

Additional file 2 **The mouse microarray data set of microarray data set of white adipose tissue (WAT) and the microarray data set of *A. thaliana *** **and the flowering time.** Click here for file
